# Chronic pain patients with possible co-morbid post-traumatic stress disorder admitted to multidisciplinary pain rehabilitation—a 1-year cohort study

**DOI:** 10.3402/ejpt.v5.23235

**Published:** 2014-08-08

**Authors:** Tonny Elmose Andersen, Lou-Ann Christensen Andersen, Per Grünwald Andersen

**Affiliations:** 1 Department of Psychology, University of Southern Denmark, Odense M, Denmark; 2 Department of Medicine, University of Southern Denmark, Odense M, Denmark; 3 Anesthesia-Intensive Care and Pain Center South, Odense University Hospital, Odense C, Denmark

**Keywords:** Post-traumatic stress, chronic pain, rehabilitation, distress, physical functioning, opioids

## Abstract

**Background:**

Although post-traumatic stress disorder (PTSD) is a common co-morbidity in chronic pain, little is known about the association between PTSD and pain in the context of chronic pain rehabilitation.

**Objective:**

The aim of the present study was two-fold: (1) to investigate the association of a possible PTSD diagnosis with symptoms of pain, physical and mental functioning, as well as the use of opioids, and (2) to compare the outcome of multidisciplinary chronic pain rehabilitation for patients with a possible PTSD diagnosis at admission with patients without PTSD at admission.

**Method:**

A consecutively referred cohort of 194 patients completed a baseline questionnaire at admission covering post-traumatic stress, pain symptoms, physical and mental functioning, as well as self-reported sleep quality and cognitive difficulties. Medication use was calculated from their medical records. A total of 95 were admitted to further multidisciplinary treatment and included in the outcome study.

**Results:**

A high prevalence of possible PTSD was found (26.3%). Patients with possible co-morbid PTSD experienced significantly poorer general and mental health, poorer sleep quality, and more cognitive problems as well as inferior social functioning compared to patients without PTSD. Possible co-morbid PTSD did not result in higher use of opioids or sedatives. Surprisingly, possible co-morbid PTSD at admission was not associated with lower levels of symptom reduction from pre- to post-treatment.

**Conclusions:**

Possible co-morbid PTSD in chronic pain is a major problem associated with significantly poorer functioning on several domains. Nevertheless, our results indicate that pain-related symptoms could be treated with success despite possible co-morbid PTSD. However, since PTSD was only measured at admission it is not known whether rehabilitation actually reduced PTSD.

A common co-morbidity among patients referred to chronic pain rehabilitation is post-traumatic stress disorder (PTSD) (Moeller-Bertram, Keltner, & Strigo, 2012). PTSD and chronic pain share a number of symptoms and response patterns such as elevated levels of arousal, attention bias, anxiety, depression, avoidance behaviour, and limited cognitive capacity due to cognitive overload (Sharp & Harvey, 2001). For these reasons, PTSD often goes unattended if no formal screening takes place. In a recent Scandinavian study by Andersen, Andersen, Vakkala, and Elklit (2012), it was found that almost one-fourth of patients admitted to chronic pain rehabilitation also suffered from possible PTSD. An even higher prevalence rate (38.8%) has been reported in severely chronic whiplash-associated disorders (Andersen, Elklit, & Vase, 2011). The high prevalence of possible PTSD among patients referred to chronic pain rehabilitation highlights the importance of examining the association between PTSD, pain symptoms, and rehabilitation outcome.

Biological and cognitive-behavioural models outline the different mechanisms and pathways by which PTSD may impact the experience of pain, psychological distress, and level of functioning. At the biological level, PTSD may lead directly to pain sensitisation as a result of dysregulation in the stress response system (Andersen et al., 2012; McLean, 2011; Stam, 2007; Sterling & Kenardy, 2006). Furthermore, cognitive and behavioural mechanisms such as pain catastrophising, fear-avoidance beliefs, depression, and avoidance behaviours may lead to pain sensitisation as well as physical and psycho-social impairment (Asmundson, Coons, Taylor, & Katz, 2002; Lethem, Slade, Troup, & Bentley, 1983; Sharp & Harvey, 2001; Vlaeyen & Linton, 2000). In particular, catastrophising (an exaggerated negative orientation towards noxious stimuli) has been proposed as an important vulnerability to both chronic pain and PTSD (Carty, O'Donnell, Evans, Kazantzis, & Creamer, 2011).

The association between PTSD and pain as well as major impairments in physical and psycho-social functioning has been demonstrated in different patient groups. In particular, a number of veteran studies have documented high rates of co-morbidity between PTSD and chronic pain as well as extensive opioid and sedative use when these two disorders co-occur (Seal et al., 2012). In fact, pain is the most common physical symptom reported by veterans suffering from PTSD (McFarlane, Atchison, Rafalowicz, & Papay, 1994; Shipherd et al., 2007). In civilian, medical populations, PTSD is associated with higher levels of pain, disability, psychological distress, and low social functioning (Clapp, Beck, Palyo, & Grant, 2008; Phifer et al., 2011). Other common symptoms associated with both disorders include sleep disturbances and cognitive problems (Dick, Eccleston, & Crombez, 2004; Lamarche & De Koninck, 2007; Lautenbacher, Kundermann, & Krieg, 2006; Smith & Haythornthwaite, 2004). Moreover, approximately 80% of patients with PTSD also meet the criteria for at least one other psychiatric diagnosis (Sharp, 2004); usually major depressive disorder, anxiety disorder, substance abuse disorder, or somatoform disorder (Brady, 1997).

Substance abuse disorders are commonly reported among patients suffering from PTSD (Kessler, Sonnega, Bromet, Hughes, & Nelson, 1995). PTSD may, therefore, increase the risk of opioid abuse among chronic pain patients. In a recent study, Phifer and colleagues (2011) examined the role of PTSD on chronic pain and pain medication. A sample of 647 general hospital patients were analysed for the experience of trauma and PTSD-related symptoms. PTSD symptoms were found to be positively associated with the use of opioids for the purpose of pain relief. Schwartz and colleagues (2006) have reported similar findings in a sample of 173 African American outpatients recruited from a mental health centre. Both samples were recruited in low socio-economic urban populations.

## Outcome rehabilitation

According to the mutual maintenance model (Sharp & Harvey, 2001), PTSD and chronic pain are mutually maintaining disorders. Thus, interventions need to break the vicious cycle maintaining both disorders. Sharp (2004) proposes that aiming to reduce cognitive and behavioural avoidance as well as to increase activity levels should be considered as important focus points in the rehabilitation of both disorders. These aims are often among the primary goals featured in chronic pain rehabilitation. For instance, pacing/activity scheduling and in vivo exposure are commonly used in chronic pain rehabilitation. The context of pain rehabilitation may, therefore, be the ideal setting for the treatment of both chronic pain and PTSD. However, only few studies have examined outcome of chronic pain rehabilitation among pain patients with co-morbid PTSD, and most studies are limited to accident-related PTSD and pain (Beck, Coffey, Foy, Keane, & Blanchard, 2009; Dunne, Kenardy, & Sterling, 2012; Stålnacke & Östman, 2010; Wald, Taylor, Chiri, & Sica, 2010). In the only existing randomised study to date, Dunne and colleagues (2012) assessed a trauma focused, cognitive-behavioural therapy programme for PTSD in the context of chronic whiplash. The intervention was found to be clinically significant in that it improved both PTSD and pain-related symptomatology. Other studies have documented a reduction in PTSD symptoms but not in pain symptoms (Beck et al., 2009; Wald et al., 2010), with the exception of Stålnacke and Östman (2010) who found a significant reduction in both domains.

## Aim and hypotheses

The aim of the present study was two-fold: (1) to investigate the association of a possible PTSD diagnosis with symptoms of pain, physical, and mental functioning as well as on the use of opioids, and (2) to compare the outcome of multidisciplinary chronic pain rehabilitation for patients with a possible PTSD diagnosis at admission with patients without PTSD at admission.

### Hypotheses

First, it was hypothesised that chronic pain patients with possible co-morbid PTSD at admission to chronic pain rehabilitation experienced lower levels of physical and mental functioning compared to patients without PTSD at admission. Second, a higher use of opioids and sedatives was expected to be reported among patients with possible co-morbid PTSD at admission compared to patients without PTSD. Finally, it was expected that patients with possible co-morbid PTSD at admission experienced poorer sleep quality and more cognitive problems compared to patients without PTSD. Given that very few studies have actually compared outcome of multidisciplinary pain rehabilitation for patients with and without PTSD and that they tend to report mixed findings, the aforementioned hypotheses are the first to put forward.

## Method

### Participants

All of the patients in this study were consecutively referred to multidisciplinary pain rehabilitation between the period of May 2009 to May 2010, and they were thoroughly assessed by a multidisciplinary team before admission. A total of 194 patients who were assessed completed the PTSD measures as well as the various outcome measures featured in this study: pain; physical and mental functioning; use of opioids, antidepressants, benzodiazepines and sleeping pills; sleep quality and cognitive problems. The total sample was used for the purposes of investigating the primary aim of this study and to test the hypotheses. Demographic details and sample characteristics are presented in Table 1.

**Table 1:** Demographic details and characteristics of the sample

Variable		%
Gender	Female	65.3
Marital status	Married/de facto	73.1
	Single	26.9
Education	Basic (mandatory)	29.2
	Further education	36.1
	Higher education	34.8
Current employment	Full time	8.6
	Part time	10.4
	Retired	43.5
	Sick leave	13.5
	Non-employed	22.7
	Study	1.2
Primary pain location	Headache	9.1
	Neck	26.9
	Upper back	6.8
	Lower back	22.8
	Chronic widespread pain	10.0
	Chronic regional pain	2.3
	Visceral	4.1
	Not specified	18.0
Chronicity in years	Mean (SD)	10.85 (11.47)
Age in years	Mean (SD)	48.24 (13.93)

*Note*: Chronicity in years=years with chronic pain, without pain-free episodes lasting >3 months.

Only 95 patients were admitted to further multidisciplinary treatment. In total, 29 patients were discharged after their first visit with the physician, either because the waiting list for further multidisciplinary treatment in the pain centre was too long or because they chose another treatment outside the pain centre. The other 70 patients only received medical treatment. These 99 patients (the 29+70) did not differ significantly (*p*≥0.062) from the total sample with regard to age, gender, or years with chronic pain or on any of the outcome variables (PTSD symptoms, level of pain, physical and mental functioning). However, the 95 patients admitted for further multidisciplinary treatment in the pain centre were significantly (*p*=0.024) younger than the total sample (mean age years 41.3 vs. 48.24) and have had pain for significantly (*p*=0.047) fewer years (mean number of years with pain 8.8 vs. 10.85). Also, a higher prevalence of possible PTSD (30.4%) was found among patients admitted to further multidisciplinary treatment in the pain centre. However, the difference in prevalence was not statistically significant (*p=*0.300). Only 46 patients returned the final questionnaire administered post-treatment. The responders did not differ significantly from the non-responders on any variables (*p*≥0.095). This final sample (n=46) was used for the purpose of investigating the secondary aim of this study, that is, to compare outcome of multidisciplinary chronic pain rehabilitation for patients with a possible PTSD diagnosis at admission with patients without PTSD at admission.

The service featured in this study is a specialist, multidisciplinary pain rehabilitation centre that receives referrals from the municipal health care system in Denmark. The team of specialists includes anaesthesiologists, nurses, physiotherapists, social workers, as well as clinical psychologists. All of the patients in this study were assessed by this team of specialists and were admitted to the optimal combination of multidisciplinary treatment according to their individual needs. Exclusion criteria included inability to speak Danish as well as current major psychiatric disorders and substance abuse disorder, as outlined in the DSM-IV (American Psychiatric Association, 1994). Moreover, patients had to have been given a diagnosis of a non-malign pain condition lasting for at least 6 months. The review board of Southern Denmark approved the research protocol. All of the patients volunteered freely to participate in this study, and written consent was provided at treatment admission.

### Measures

Physical and mental health was assessed using the Medical Outcomes Study 36 Item Short-Form Health Survey (SF-36; Ware & Sherbourne, 1992). The SF-36 is a well-validated, health status instrument that measures the components of physical and mental health on eight different dimensions. The physical health component comprises physical functioning, role of physical bodily pain, and general health. The mental health component comprises vitality, social functioning, role-emotional, and mental health. The subscales aggregate 2–10 items each. The scale has been shown to be psychometrically sound in general as well as in specific populations such as chronic pain populations (Turner-Bowker et al., 2002). The reliability of the eight scales and the two summary measures (mental and physical health) has been estimated using both internal consistency and test–retest methods. Most reliability measures have exceeded 0.80. For the two summary measures, scores usually exceed 0.90 (Ware et al., 1998).

The Harvard Trauma Questionnaire part IV (HTQ; Mollica, Caspi-Yavin, Bollini, & Truong, 1992) was used to measure the presence of PTSD. The HTQ consists of 17 items featuring a 4-point Likert scale (1=not at all to 4=very often). The 17 items relate to the following core symptom clusters of PTSD as outlined in DSM-IV (American Psychiatric Association, 1994): avoidance (7 items, α=0.88), re-experiencing (5 items, α=0.88), and hyper vigilance (5 items, α=0.88). Patients were given a possible PTSD diagnosis if they met all the DSM-IV criteria for PTSD. They had to report at least one re-experiencing symptom, three avoidance symptoms, and two hyper-arousal symptoms. Items were deemed to be positively endorsed if scores were ≥3. It has previously been reported that the HTQ self-report measure of PTSD has an 88% concordance rate with interview-based estimates of PTSD (Mollica et al., 1992). The internal consistency of the HTQ, as measured by Cronbach's alpha, was excellent (total α=0.95). Before completing the HTQ, patients were asked to identify significant traumatic stressors from a 20-item list comprising experiences of both direct and indirect exposure to traumatic events. Moreover, patients were asked to identify the event that they considered to be the primary traumatic event. The items were based on a variety of experiences featured in the DSM-IV diagnostic criteria for traumatic exposures (American Psychiatric Association, 1994).

Medication use was registered according to several categories based on information obtained at the initial physical examination upon referral. Medication use for each category of drug (opioids, antidepressants, benzodiazepines, and sleeping pills) was coded as either present (1) or absent (0). Opioid use was measured according to intake of mg morphine per day.

Patients were asked to rate their general sleep quality during the past month on a 5-point numerical rating scale (0=best possible sleep quality, 5=worst possible sleep quality). Experienced cognitive problems (concentration and memory) were measured using a simple self-report scale from 0–5. (0=no cognitive problems, 5=severe cognitive problems).

### Statistical analyses

Descriptive analyses and demographic details are presented as percentages, means, and standard deviations. Outcome scores were calculated as transformed scores ranging from 0–100, with higher scores indicating better results. Missing values for the SF-36 subscales were approximated according to the SF-36 guidelines, by using the mean values of the remaining items if at least half of the corresponding items were valid.

At admission, a comparison regarding scores on outcome variables (SF-36, and mg opioids used per day) was made between patients with and without possible co-morbid PTSD. This was done using independent, 2-tailed *t*-tests with Holm's adjustment for multiple testing. Adjusted *p-*values were calculated by sorting the unadjusted *p-*values in decreasing order of significance and then adjusted sequentially by multiplying the first (lowest) unadjusted *p-*value by k (number of conditions), the next by k-1, the next by k-2, and so forth.

The single items score, sleep quality, and cognitive function was compared using Mann–Whitney tests. Also, at admission, medication use was calculated as dichotomised variables using Chi square (χ^2^) tests. Finally, repeated measures analysis of variance (ANOVA) was used to compare outcome of multidisciplinary pain rehabilitation (pre- to post-treatment scores) for patients with and without possible co-morbid PTSD. The groups were compared on the two general dimensions comprising the SF-36: the physical and mental component scales. Only these two general dimensions were used due to the small sample size of the study. It was for this same reason that covariates such as gender and age were not adjusted for. Effect sizes for *t*-tests were calculated using Cohen's *d* (Cohen, 1988). Effect sizes for ANOVA were calculated using partial eta-square. Effect sizes (*d)* were evaluated based on the following criteria: 0.2=small effect, 0.5=medium effect, and 0.8=large effect. Partial eta-squares (η^2^_p)_ were evaluated based on the following criteria: 0.01=small effect, 0.09=medium effect, and 0.25=large effect (Cohen, 1988). All statistical analyses were conducted using SPSS version 20.0.

## Results

Using the DSM-IV diagnostic criteria for PTSD, the prevalence of a possible PTSD diagnosis in the present sample was 26.3%. The most frequently reported traumatic events among PTSD cases were *having experienced a traffic accident* or *having experienced another accident*. These events accounted for one-fourth of all possible PTSD cases. The second most frequently reported traumatic events were *having experienced losing someone close* (15.7%) and *having experienced serious illness or having witnessed serious illness to someone close* (11.8%). No significant association was found between the prevalence of possible PTSD and specific pain diagnosis (*p*≥0.084). Furthermore, of those patients admitted to rehabilitation, no significant differences regarding gender, age, and number of years of having a chronic pain diagnosis were found between patients with and without possible co-morbid PTSD (*p* ≥0.082).

### Baseline outcomes for patients with and without possible co-morbid PTSD

As presented in Table 2, independent *t*-tests showed that, regarding the physical health component, only general health reached statistical significance (*p*=0.012), indicating that patients with possible co-morbid PTSD experienced poorer general health compared to patients without PTSD. Regarding the psycho-social outcomes, patients with possible co-morbid PTSD experienced significantly poorer mental health (*p*=0.007) as well as inferior social functioning (*p*<0.001) compared to patients without PTSD. Moreover, patients with possible co-morbid PTSD reported significantly poorer sleep quality (*M*=63.73) compared to those without PTSD (*M*=87.72), *U=*1,773, *Z*=−2.96, *p=*0.003, *r*=0.23. Finally, patients with possible co-morbid PTSD reported significantly more cognitive problems (*M*=61.74) compared to patients without PTSD (*M*=88.64), *U*=1,709, *Z*=−3.48, *p=*0.001, *r*=0.27.

**Table 2:** Independent *t*-tests for baseline outcome measures

SF-36	PTSD (*n*=51)		Non-PTSD (*n*=143)					95% CI		Cohen's
		
Subscales	*M*	*SD*	*M*	*SD*	*t* (192)	Adjusted *p*	*p*	*LL*	*UL*	*d*
PF	43.78	23.74	43.82	22.06	0.01	1.000	0.993	−8.11	8.18	0.00
RP	9.87	31.59	14.42	34.08	0.72	1.000	0.474	−7.98	17.09	0.14
BP	20.79	15.37	20.99	16.77	0.06	1.000	0.949	−5.84	6.23	0.01
GH	23.47	13.94	34.11	18.85	3.17	**0.012**	**0.002**	4.01	17.26	0.61
VT	23.78	17.20	29.42	20.64	1.56	0.605	0.121	−1.50	12.78	0.29
SF	35.67	19.28	50.98	29.53	3.75	**<0.001**	**<0.001**	7.22	23.39	0.57
RE	35.00	79.15	44.98	48.44	0.92	1.000	0.362	−11.59	31.56	0.17
MH	41.37	20.19	55.06	22.96	3.37	**0.007**	**0.001**	5.66	21.73	0.62

*Note*: Bold results=*p<*0.05. Adjusted *p*=Holm's adjusted *p-*values. PF=physical functioning, RP=role-physical, BP=bodily pain, GH=general health, VT=vitality, SF=social functioning, RE=role-emotional, MH=mental health.

### Medication use for patients with and without possible co-morbid PTSD

Categories of medication use among the two patient groups are presented in Table 3. No significant differences were found between the two patient groups regarding medication use (*p*≥0.195), the only exception being for antidepressants. Patients with possible co-morbid PTSD used significantly more antidepressants compared to patients without PTSD (*p*=0.013).

**Table 3:** Medication use by category

	All	PTSD	Non-PTSD			
				
Medications	*N=*194	*n=*51	*n=*143	χ^2^	df	*p*
Antidepressants (%)	39.4	54.9	33.8	6.14	1	**0.013**
Benzodiazepines (%)	15.0	21.6	12.7	1.68	1	0.195
Sleeping pills (%)	13.0	17.6	11.3	0.85	1	0.357
Morphine (mg)/day	*N=*194	*n=*51	*n=*143	*t*	df	*p*
Mean (SD)	61.87 (121.53)	54.67 (86.0)	69.74 (139.12)	0.73	191	0.469

*Note*: Bold results=*p<*0.05. Sleeping pills = non-benzodiazepines, only prescribed for sleeping problems.

### Comparison of physical and mental component scores

**Fig. 1:**
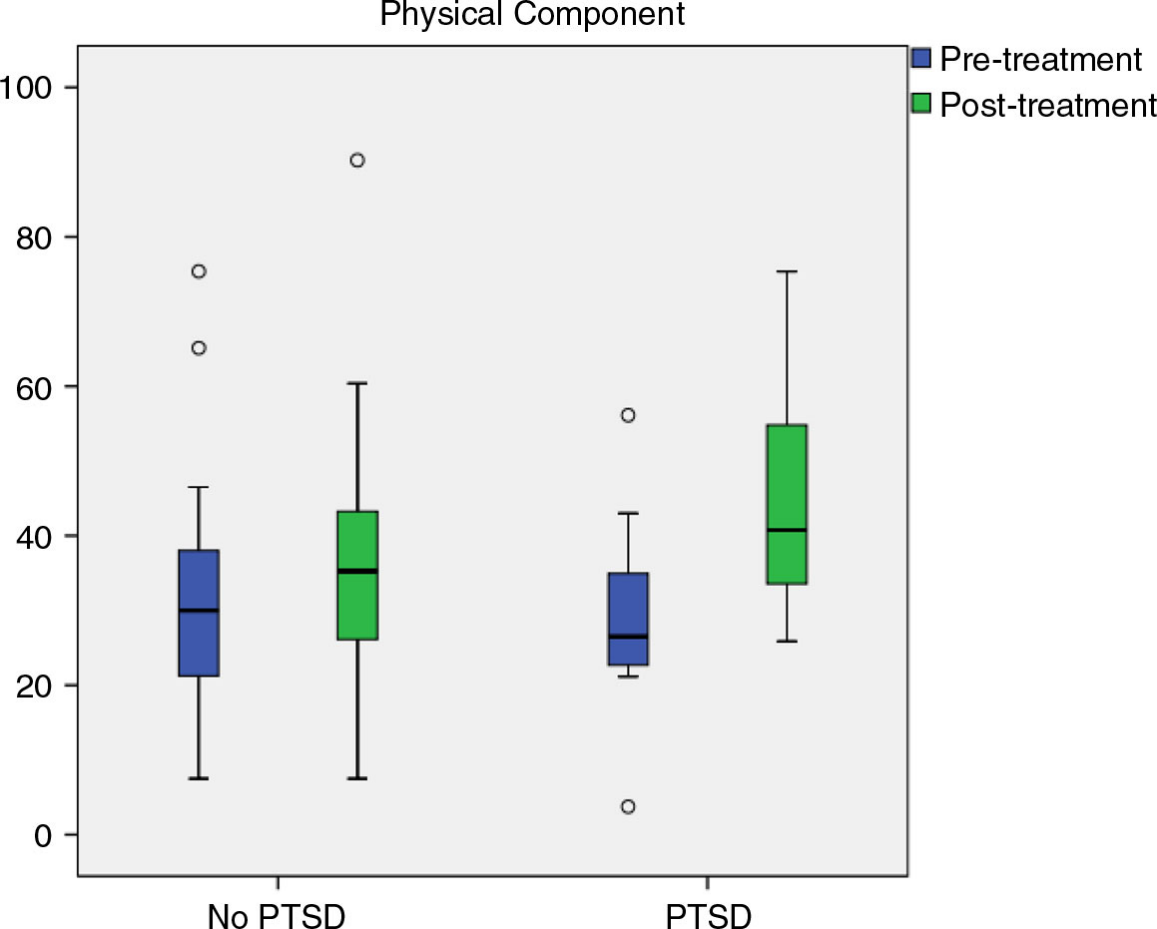
Between group comparisons for SF-36 physical component.

The results of the ANOVA showed a large reduction on the physical component scale after multidisciplinary rehabilitation (*F*(1.38)=11.89, *p*=0.001, η^2^_p_=0.24). However, no significant group difference was found in level of symptom reduction from pre- to post-treatment (*F*(1.38)=3.07, *p*=0.088, η^2^_p_=0.08) (Fig. 1).

**Fig. 2:**
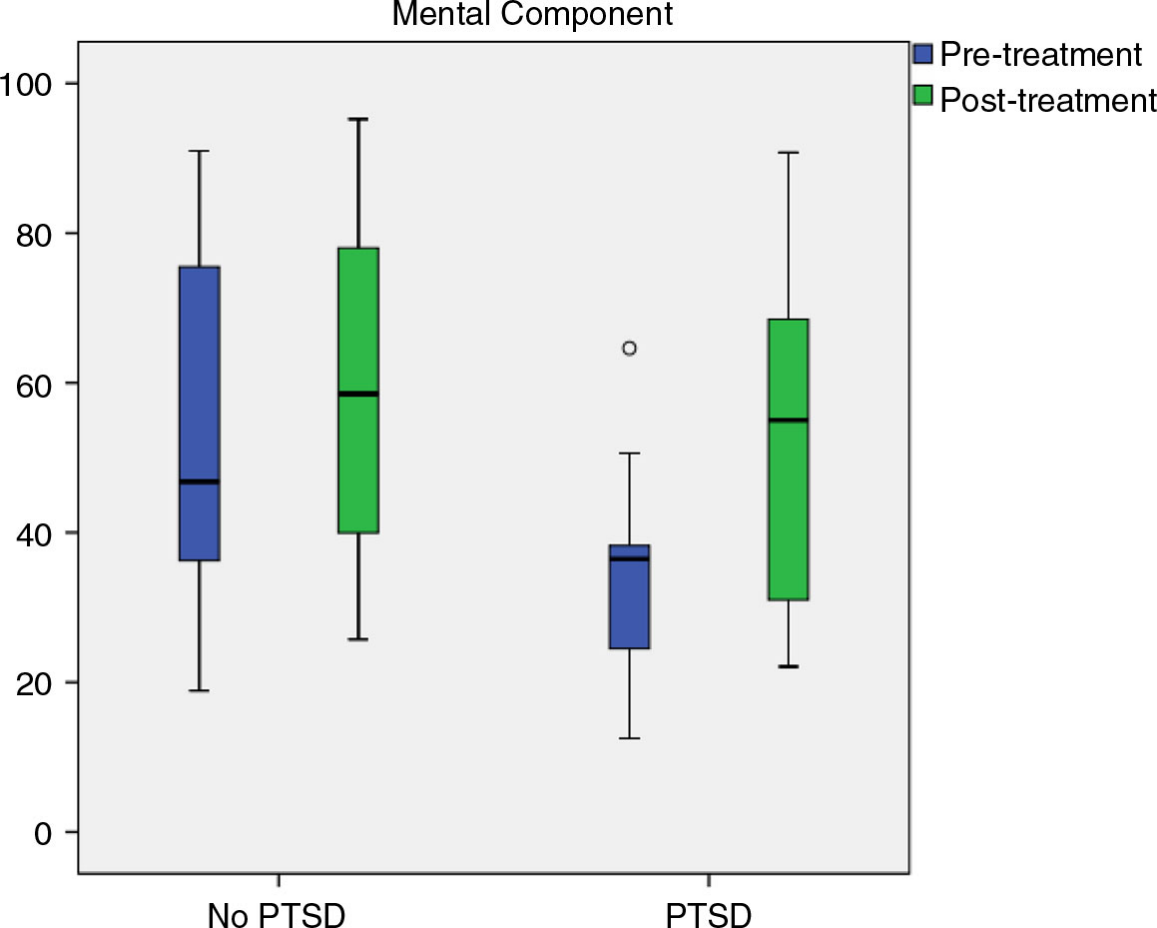
Between group comparisons for SF-36 mental component.

The results of the ANOVA showed a large reduction on the mental component scale after rehabilitation (*F*(1.44)=11.13, *p*=0.002, η^2^_p_=0.20). Moreover, there was a significant group difference on the mental component scale (*F*(1.44)=4.16, *p*=0.047, η^2^_p_=0.09). This indicates that the group with possible co-morbid PTSD achieved a larger symptom reduction from pre- to post-treatment compared with patients without co-morbid PTSD (Fig. 2).

## Discussion

Our hypothesis that chronic pain patients with possible co-morbid PTSD experience lower levels of physical and mental functioning was only partially confirmed. Surprisingly, no significant differences were found comparing levels of pain and physical functioning for those with and without possible co-morbid PTSD. Only general health was experienced as poorer in those with possible co-morbid PTSD compared to those without. Therefore, earlier findings using both civilian medical populations (Clapp et al., 2008; Phifer et al., 2011) and veterans (McFarlane et al., 1994; Shipherd et al., 2007) could not be confirmed in the present study. The negative findings may be due to the use of different population samples. In the present study, patients had, on average, suffered from chronic pain for more than 10 years. In the study by Clapp and colleagues (2008), the average number of years of suffering from chronic pain was 2 years. It is possible that patients with severe chronic pain resemble patients with chronic PTSD more than patients with less severe pain due to mutual maintenance and exacerbation of the two conditions. Moreover, while the present study included patients with various pain diagnoses, the study by Clapp and colleagues (2008) included only patients diagnosed with chronic pain after motor vehicle accidents. Furthermore, the study by Phifer and colleagues (2011) used a highly selected subsample of African American patients recruited from the waiting room at a general hospital in a low socio-economic urban area. The association between PTSD and physical functioning may, therefore, diverge for different populations, which is why it is difficult to generalise this association to the general chronic pain population. As found in other studies (Dick et al., 2004; Lamarche & De Koninck, 2007; Lautenbacher et al., 2006; Smith & Haythornthwaite, 2004), chronic pain patients with possible co-morbid PTSD reported lower mental functioning, more cognitive symptoms, and poorer sleep quality compared with their counterparts without PTSD.

Our hypothesis that chronic pain patients with possible co-morbid PTSD would report a higher use of opioids and sedatives compared to those without PTSD was not confirmed. This is different from the results of Phifer and colleagues (2011) and Schwartz and colleagues (2006), who found that pain patients with co-morbid PTSD use more opioids for the purposes of pain relief than pain patients without PTSD. The negative findings of the present study may, again, be the result of a sampling bias; the studies by Phifer and colleagues (2011) and Schwartz and colleagues (2006) recruited patients from low socio-economic urban areas. Furthermore, differences in the referral process and the social health care system may also have affected the results. In the present study, sampling bias may have taken place early in the visitation procedure to the multidisciplinary pain centre. One-fourth of all referred patients were rejected administratively at the visitation before admission to the pain centre. The primary causes for rejection were lack of a clear diagnosis or that the pain condition was not stationary. Also, current major psychiatric disorders and distinct substance abuse was exclusion criteria for admission. Knowing that PTSD and substance abuse disorders often co-occur as a result of self-medication (Khantzian, 2003; Swendsen et al., 2010) exclusion of patients with distinct substance abuse may have resulted in the negative findings regarding the association between PTSD and the use of opioids and sedatives. It was found that chronic pain patients with possible co-morbid PTSD used more antidepressants than patients without PTSD, which may be a reflection of the elevated levels of psychological distress associated with PTSD among this patient group. In agreement with (Dick, Eccleston, & Crombez, 2004; Lamarche & De Koninck, 2007; Lautenbacher, Kundermann, & Krieg, 2006; Smith & Haythornthwaite, 2004), the third hypothesis of this study was confirmed; chronic pain patients with possible co-morbid PTSD experienced significantly poorer sleep quality and more cognitive problems compared to patients without PTSD.

When pain and PTSD symptoms co-occur, clinicians are likely to be required to modify their treatment protocols accordingly, for example, by adapting existing cognitive-behavioural therapy programmes in order to address both conditions. To date, there is no systematic empirical data, which support the treatment of both conditions separately or simultaneously (Asmundson et al., 2002). Although, the follow-up data of the present study is limited by a small sample size and no adjustment for treatment allocation, the results *do* indicate that pain-related symptoms can be treated with success despite possible co-morbid PTSD at admission to multidisciplinary chronic pain rehabilitation. However, since PTSD symptoms were only measured at admission, it is not known whether rehabilitation actually reduced the PTSD symptoms. This is in agreement with Stålnacke and Östman (2010). Although PTSD was not targeted directly during the multidisciplinary pain rehabilitation featured in this study, cognitive-behavioural techniques were used to target catastrophising and symptoms of anxiety and depression. Moreover, functional restoration was a primary goal, which may have affected behavioural avoidance and anxiety in general. The results are implicitly in agreement with Sharp and Harvey (2001), who suggest that exposure strategies for PTSD and pain avoidance can be combined. By doing so, patients with pain and PTSD can, simultaneously, confront and overcome their fears and engage in activities and exercises from which they will benefit physically (Sharp, 2004).

## Strengths and limitations

A major limitation associated with the present study is that PTSD was only measured at baseline. For this reason, it is not known whether rehabilitation actually reduced PTSD symptoms or how chronic pain and PTSD may have mutually maintained each other. Also, the absence of a structured clinical interview for PTSD is a limitation. Moreover, because of the small sample size of the follow-up part of the study and the overall multidisciplinary approach to all patients, the present study was unable to determine which parts of the interventions may have contained the so-called “active” ingredients. In this regard, a randomised controlled study investigating the impact of co-morbid PTSD on outcome of pain rehabilitation should be carried out. Despite these limitations, the study also has several strengths. A major strength is that the study used a cohort of patients consecutively referred to multidisciplinary chronic pain rehabilitation. The total sample of patients (n=194) is believed to be representative of chronic pain patients admitted to multidisciplinary chronic pain rehabilitation, which is essential given that very few studies have investigated co-morbid chronic pain and PTSD in the context of multidisciplinary pain rehabilitation. However, the sample may not be representative of chronic pain patients in general.

## Conclusion

Future studies should investigate the impact of co-morbid PTSD on chronic pain rehabilitation in the context of a randomised controlled trial comparing different treatment conditions. In addition, it is extremely relevant to examine whether the outcome of chronic pain rehabilitation is influenced by the aetiology of the pain condition and trauma. When the onset of pain is related to a traumatic event, as it is in many traffic accidents, the mutually maintaining mechanisms of the two disorders may be different for patients suffering from co-morbid PTSD unrelated to the onset of pain. When the onset of pain is associated with a traumatic event, pain may serve as a reminder of the traumatic event leading to re-experiencing (Asmundson et al., 2002). Unfortunately, the present study was unable to determine whether the onset of pain was related to PTSD. This may partly explain our negative findings regarding the hypothesised association among possible PTSD, physical functioning, and pain. The negative findings may be related to the fact that the primary traumatic event experienced by patients was accident related. It is possible that more interpersonally related traumas may be more complex to treat in the context of chronic pain rehabilitation where the primary goal is activity restoration via pacing and activity scheduling. Also, patients with a co-morbid substance abuse disorder in addition to PTSD may need more specialised treatment in addition to multidisciplinary pain rehabilitation.
